# Microplastic Contamination in Urban, Farmland and Desert Environments along a Highway in Southern Xinjiang, China

**DOI:** 10.3390/ijerph19158890

**Published:** 2022-07-22

**Authors:** Wenfeng Li, Shuzhi Wang, Rehemanjiang Wufuer, Jia Duo, Xiangliang Pan

**Affiliations:** 1 National Engineering Technology Research Center for Desert-Oasis Ecological Construction, Xinjiang Institute of Ecology and Geography, Chinese Academy of Sciences, 818 South Beijing Road, Urumqi 830011, China; liwf86@ms.xjb.ac.cn (W.L.); wangshuzhi.1234@163.com (S.W.); duojia2017@ms.xjb.ac.cn (J.D.); 2 Xinjiang Key Laboratory of Environmental Pollution and Bioremediation, Xinjiang Institute of Ecology and Geography, Chinese Academy of Sciences, Urumqi 830011, China; 3 Key Laboratory of Microbial Technology for Industrial Pollution Control of Zhejiang Province, College of Environment, Zhejiang University of Technology, Hangzhou 310014, China

**Keywords:** microplastics, distribution, shape categories, abundance, southern Xinjiang

## Abstract

The different types of microplastics (MPs), including debris, fibers, particles, foams, films and others, have become a global environmental problem. However, there is still a lack of research and understanding of the pollution characteristics and main causes of MPs in the arid region of Xinjiang, China. In this survey, we focused on the occurrence and distribution of MPs in urban, farmland and desert areas along a highway in the survey area. Our results showed that the main types of MPs were polypropylene (PP) flakes, polyethylene (PE) films and both PE and PP fragments and fibers. The abundance levels of MPs in street dust of Korla, Alar and Hotan districts equaled 804, 307 and 1526 particles kg^−1^, respectively, and were positively correlated with the urban population. In farmland areas, there were only two types of MPs (films and fibers), of which the film particles dominated and accounted for 91% of the total on the average. The highest abundance rate of MPs reached 7292 particles kg^−1^ in the desert area along the highway. The minimum microplastic particle sizes were 51.8 ± 2.2 μm in urban street dust samples, 54.2 ± 5.3 μm in farmland soil samples and 67.8 ± 8.4 μm in samples from along the desert highway. Particle sizes < 500 μm were most common and accounted for 48–91% of the total in our survey. The abundance and shape distribution of the MPs were closely related to the different types of human activities.

## 1. Introduction

Plastic materials and products are widely used in daily life and in industry. In 2018, the global plastic production volume reached nearly 360 million tons, with China (30%), Europe (17%) and North America (18%) producing the most raw materials [1]. The mass production and widespread use of plastics has led to the ubiquity of plastic debris in the terrestrial environment, which will have a profound impact on human health and the global environment. Thompson et al. defined microplastics (MPs) as plastic particles < 5 mm in size [2]. Because plastic products are highly resistant to degradation, it is expected that about 630 million tons of plastic waste could be produced and approximately 120 million tons of MPs could be accumulated on earth by 2050 [3].

Due to their wide distribution, MPs are likely to act as vectors for various man-made contaminants that transfer into terrestrial and aquatic environments, posing a greater threat to the ecological security and sustainable development of human society. Intensive anthropogenic activities have resulted in large amounts of plastic debris entering the terrestrial ecosystem. MPs may be produced with further breakage and degradation, and most of these emissions occur in urban and residential areas, such as houses, commercial areas and industrial areas [4]. Numerous reports have been published on the occurrence of MPs in the environment that originate from fertilizers [5], polyethylene (PE) mulch [6], urban wastewater [7], street litter and illegal waste discharge [8], tire abrasion [9] and the long-range transport of atmospheric particles [10]. Researchers have paid attention to MPs and have warned of their dangers in soil and terrestrial eco-systems.

Despite knowing that MPs are widespread in terrestrial environments due to human activities [11], quantitative studies of MPs in arid regions have been lacking [12]. Different frequencies and types of human activities may lead to significant differences in the categories, quantities, sizes and distributions of MPs. However, the characteristics and distribution of MPs have attracted less attention in the arid interiors, especially in desert areas [13].

Southern Xinjiang has a typical arid climate in northwest China, with an average annual precipitation rate of 150 mm. Most of the crops are covered with film during growth, and particularly almost all cotton plants are film-mulched [14]. It has been reported that residual mulch is the main source of MPs on land [15]. Korla, Alar and Hetian are important cities in southern Xinjiang with relatively concentrated populations and relatively developed economies. Due to the long-term human activities and irrational disposal of plastic products, there may be serious MP pollution in the surrounding environment. The desert highway from Alar City to Hotan City is 424 km long, greatly promoting the economic and social prosperity of southern Xinjiang. Because of the fluidity of the Taklimakan Desert, highway builders and researchers have built mechanical protection systems on both sides of the road, using grass grids and plastic sand barriers. Due to the meteorological characteristics of extreme drought, high temperature and strong ultraviolet radiation in the desert, the continuous damage from the plastic sand barrier might have caused microplastic pollution in the surrounding environments.

However, there is no relevant report so far and we lack data support for the above hypothesis. It is, therefore, important to understand the current status of MPs in the cities, farmlands and deserts along the highways in southern Xinjiang, China. The purpose of this survey was to evaluate MP pollution in cities, farmlands and deserts along the highways in southern Xinjiang, China, by examining the MP distribution characteristics such as the shape, size, composition and abundance. The effects of human activities and meteorological factors on the MP pollution in these areas were analyzed. In addition, possible environmental and health risks were assessed. The research results may help local authorities to formulate preventive measures and relevant laws and regulations for microplastic pollution, and could provide a certain data reference to promote local environmentally sustainable development.

## 2. Materials and Methods

### 2.1. Sampling

**Figure 1:**
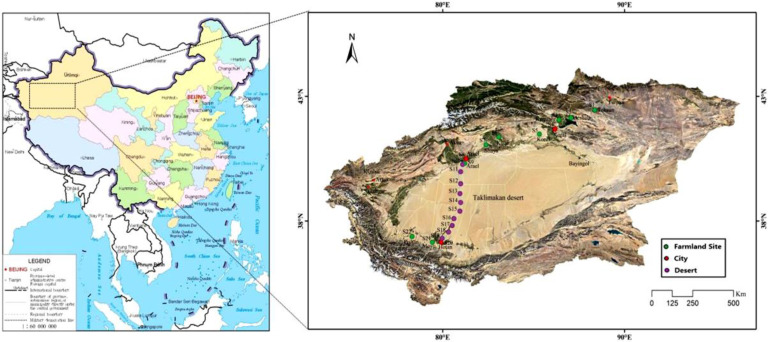
Sketch map showing the sampling sites in southern Xinjiang (drawing number: Gs (2019) 1654).

The survey area spanned the entire southern Xinjiang, passing through the regions of Toksun, Korla and Arael, traversing the Taklamakan desert and ending in the Hotan region. Fifteen dust samples from three major cities, 45 soil samples from 9 counties and 10 desert samples from the Taklimakan desert along the desert highway were collected during the survey in April, 2020. The dust samples were taken from the streets of Korla, Arael and Hotan (S4, S8 and S20). The soil sample sites were distributed in 9 counties along the highway (such as S1, Toksun; S2, Heshuo; S3, Karasahr; S5, Korla; S6, Kuqa; S7, Shaya; S9, Alaer; S21, Moyu; S22, Pishan). The desert sample sites were distributed within five meters on both sides of the highway (S10–S19), including 3 garbage stacking sites (S11, S16, S19) and 3 sand fixation sites (S15, S17, S18). The map and coordinates of the sampling sites are shown in Figure 1 and Table S1.

Considering the influence of meteorological and human factors, we used different sampling methods for different types of samples. Here, 500 g of street dust samples was collected from the streets of each city’s different functional zones, such as the street surfaces adjacent to the curbs in each city’s business district, residential area, school and main stem and business districts, and then mixed well before being carefully swept into a fabric pocket with a metal plate and a wooden brush. Five replicate soil samples from each site were taken randomly using a multipoint mixed method. Each soil sample was collected in a 0.1 × 0.1 m^2^ quadrant to a depth of 0.2 m using a narrow stainless steel shovel. The mass of each sample was approximately 20 kg. The samples were well mixed and transferred to cloth bags. The sand samples were taken from roughly 5 cm of the desert surface (1 × 1 m^2^ square area) with a clean stainless steel shovel using the coastal beach sampling method. Three duplicate samples were collected randomly from each site and pooled as one. The mass of each sample was about 4 kg.

The street dust, soil and sand samples were air-dried in the laboratory to avoid polluting the samples, then passed through a 5 mm stainless steel sieve to remove larger debris such as stones, root residue and large pieces of garbage. The MP particle abundance was determined based on the dry weight of the sediment samples.

### 2.2. Extraction of MPs 

Each collected sample was homogenized by shaking and leached using pressurized flow water until the leaching water became clear. The residue on the stainless steel sieve (300 mesh, *Φ* = 0.050 mm) was collected in glass dishes, washed with ultrapure water and floated using a saturated sodium chloride solution (1.12 g cm^−3^). The supernatant of each flotation was decanted into a 500 mL glass beaker and filtered onto 10–20 μm quantitative paper (*Φ* = 9 cm, Xinxing, China) under vacuum conditions. Then, the organic matter in the MPs was treated with 30% H_2_O_2_ at room temperature for 48 h via digestion. The digestion solutions were filtered through a 0.45 μm filter (*Φ* = 47 mm, Whatman). Finally, the filter membrane was washed with deionized water and carefully transferred to a clean Petri dish using washed metallic tweezers before being covered and dried in a drying oven at 50–60 °C for 12 h.

### 2.3. Observation and Identification of MPs

The samples were observed under a multifunctional magnifying lamp (German PDOK, 10×, Guangdong, China) and a microscope (Olympas, IX81, Olympus Corporation, Tokyo, Japan). Particles that were visually recognized or suspected to be “plastics” were transferred to clean, smooth and black cardboards and classified according to their shapes [16]. All particles transferred to the cardboard were photographed using a Nikon D3200 digital camera for particle counting and size measurements. The MPs were classified into six categories according to their size (<0.5 mm, 0.5–1.0 mm, 1.0–2.0 mm, 2.0–3.0 mm, 3.0–4.0 mm and 4.0–5.0 mm).

A set of representative microplastic particles (>0.15 mm) from each site was selected to identify their chemical components using the ATR-FTIR method [17]. ATR-FTIR spectra were recorded on a Bruker Tensor 27 FTIR spectrometer with Pike Miracle ATR accessories. We followed the European Commission’s Marine Strategy Framework Directive recommendations that at least 10% of recorded fragments and filaments should be analyzed by FTIR [18].

### 2.4. Data Analysis

All results for MP abundances were expressed as the number of microplastic particles per dry mass soil (particles kg^−^^1^). The particle counting and size measurements were performed using Nano Measurer 1.2 software (Nano Measurer 1.02, 2008 Jie Xu, China). The data analysis was performed using WPS Office 2019 and OriginPro 8.5 (OriginLab Corporation, Northampton, MA, USA). The spatial distribution of MPs was mapped using ArcGIS10.2 (ESRI, Redlands, CA, USA).

### 2.5. Quality Assurance and Quality Control

In order to guarantee the accuracy of our data, a series of quality assurance and quality control (QA/QC) measures were taken during the process from field sampling to laboratory analysis [19]. Stainless steel or glass materials were used during sampling, processing and analysis. The stainless steel scoop was washed with deionized water before each sampling. All dishes, beakers and flasks used during the experiment were washed three times with deionized water and covered with aluminum foil after each step. The MPs were separated and enumerated in clean and separate rooms and stored in closed spaces to avoid pollution from airborne MPs. All liquids used in the experiment were filtered through the 0.45 μm filter (GF/B *Φ* = 47 mm, Whatman). The least significant differences (LSD) test was performed for multiple mean comparisons at *p* < 0.05. The blank controls were produced using the same processing methods as those applied to the field samples and in laboratory process, and the recovery rate was 92.6 ± 3.5%.

## 3. Results

### 3.1. The Abundance of MPs in Different Sampling Sites

**Figure 2:**
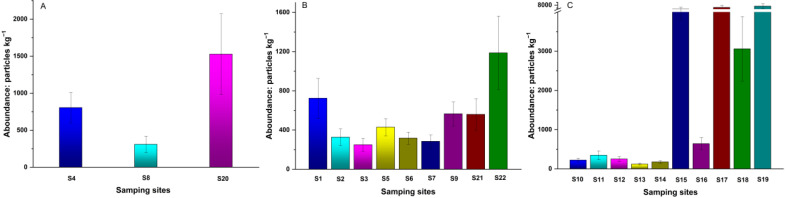
MP abundance levels in different sampling sites: (**A**) city; (**B**) farmland; (**C**) Taklimakan Desert.

Figure 2 provides the abundance of MPs in different sampling sites in the cities and croplands and in the Taklamakan dessert along the highway. The numbers of MPs in the dust samples collected in the streets of Korla, Alar and Hotan equaled 804, 307 and 1526 particles kg^−1^, respectively (Figure 2A). The contents of MPs in the soil samples from the 10 counties ranged from 284 to 1187 particles∙kg^−1^, with Pishan county’s soil having the highest MPs abundance (1187 particles kg^−1^), followed by Toksun county soil (724 particles kg^−1^, Figure 2B). The abundance of MPs in the desert sampling sites fluctuated considerably, with the highest level reaching 7292 particles kg^−1^ and the lowest reaching 119 particles kg^−1^ (Figure 2C).

### 3.2. Shape Categories and Compositions

**Figure 3:**
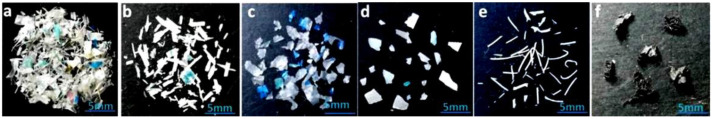
Different shapes of partial microplastic samples collected from the urban streets, farmland soils and desert areas along the highway in southern Xinjiang, China: (**a**) mixed MPs; (**b**) flakes; (**c**) films; (**d**) fragments; (**e**) fibers; (**f**) rubbers.

Based on their morphological characteristics, the plastic or microplastic samples were generally divided into flakes, films, fragments, fibers and rubber, and whether they occurred in the city, farmland or desert (Figure 3 and Figure S1). Figure 3a shows the mixed morphological shapes of MPs found in this survey in the cities, croplands and desert areas. The flakes (Figure 3b) were flat sheets of various plastic woven bags. The films (Figure 3c) were thin, soft, transparent sheets. The fragments (Figure 3d) were mainly a kind of irregular, hard plastic debris. The fibers (Figure 3e) in the samples were mostly long, curly remnants of dustproof materials and the rubber particles (Figure 3f) were usually black and irregular shapes. The main polymer types were polypropylene (PP) flakes, polyethylene (PE) films and both PE and PP fragments and fibers (Figure S2). It is worth noting that rubbers made from poly(styrene-co-butadiene) (PSB) were identified in this research.

**Figure 4:**
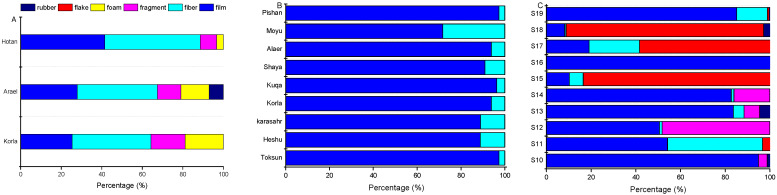
Compositions of different MP shape categories in cities (**A**), farmlands (**B**) and deserts along the highway (**C**) in southern Xinjiang, China.

Figure 4 shows the distribution patterns of different MP shapes in the urban streets, farmlands and desert areas along the highway. It can be seen from Figure 4A that fibers and films were the most common shapes of MPs in urban streets, the percentages ranging from 38.9% to 47.1% and from 25.4% to 41.5%, respectively. Among them, the proportions of fibers and films were the highest in Hotan, reaching up to 47.1% and 41.5%, respectively; followed by Aral and Korla with 39.5% and 27.9% and 38.9% and 25.4%, respectively. The proportions of foam and fragments were 18.9%, 14% and 3.3% and 16.8%, 11.6% and 8.1% for Korla, Arael and Hotan, respectively. Rubber was found only in Arael at a rate of 7%, while flakes did not appear in any of these 3 urban street samples. As shown in Figure 4B, only film and fiber MPs were found in the soil samples, with film MPs being the dominant portion ranging from 71.7% to 97.3% (91% on average), while fiber only accounted for 2.7–28.3% (9.0% on average). Figure 4C shows that the film was widespread along the desert highway, with ratios varying from 8.2 to 100%. The flakes were the dominant component in sampling sites S15, S17 and S18 (83.6%, 58.5% and 88.2%), while the highest percentages of fragments and fibers were present in S12 and S11 sampling sites (48.1% and 42.5%). The rubber only appeared in sampling sites S13, S18 and S10, with very low percentages (4.7%, 2.9% and 1.3%), while the foam was not present in any sampling sites along the desert highway.

### 3.3. Size Distribution of MPs

**Figure 5:**
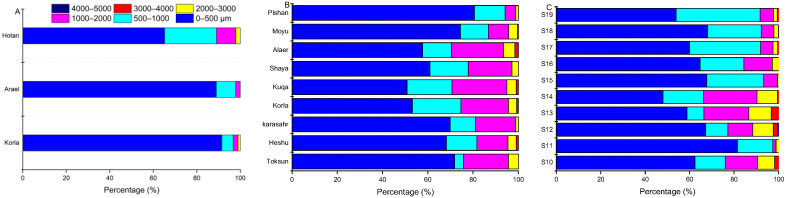
Size distribution of MPs: (**A**) urban street dust sampling sites; (**B**) farmland soil sampling sites; (**C**) deserts sampling sites along the highway.

Figure 5 shows the size distribution patterns of MPs in cities (Figure 5A), farmlands (Figure 5B) and deserts along the highway (Figure 5C) in southern Xinjiang, China. As shown in Figure 5, in general, the contents of the different particle sizes showed a decreasing trend after the increase in particle size. The MPs with particle sizes < 500 μm were dominant in all sampling sites, with the rates varying from 48.0% to 91.5%. The particle sizes of 500–1000 μm and 1000–2000 μm appeared in all sampling sites, with the percentages varying from 5.21 to 37.74 μm and from 1.45 to 24.29 μm, respectively. The particle sizes of 2000–3000 μm were present in all sampling sites except the Areal street dust sample, with a lower percentage range of 0.46–10.08 μm. The particle sizes of 3000–5000 μm were only present in several sampling sites with very low numbers (<5%). In different types of sampling sites, the mean percentage of particles <500 μm for MPs in cities was 81.90%, which was significantly higher (*p* < 0.05) than the mean percentages in farmlands (65.27%) and desert sampling sites (63.23%). Comparatively, the mean percentages of particles measuring 500–1000 μm and 1000–2000 μm in cities were 12.67% and 4.44%, respectively, which were significantly lower than the mean percentages in farmlands (13.94% and 16.96%, respectively) and desert sampling sites (20.43% and 10.78%, respectively). Additionally, our survey found that the minimum microplastic size was 51.8 ± 2.2 μm in urban street dust samples, 54.2 ± 5.3 μm in farmland soil samples and 67.8 ± 8.4 μm in samples along the desert highway.

## 4. Discussion

### 4.1. Possible Sources

Different human activities are the most important sources among the many other sources that produce plastic waste. The direct sources are mainly human life, industrial production and agricultural aquaculture, while the indirect sources mainly include landfill decomposition, sewage plant discharge and sludge utilization [20]. Once in the environment, the weathering process slowly breaks these products down and produces large amounts of MPs.

Plastic products are widely used in household products, express industry, engineering and packaging and automotive industries in different human activities [21]. Agricultural production activities produce a large amount of plastic waste, such as plastic packaging, fertilizer and woven bags, but the main source of plastic waste is low-recycling film [22]. However, there is still a lack of research on the sources of MPs in desert areas.

Film MPs are mainly attributed to plastic packaging in the urban and desert areas and to mulching in farmland [23]. Due to the lack of an efficient recovery mechanism for residual film, widespread residual plastic film use has been the main source of film MPs in arid and semi-arid agricultural regions. Irrationally discarded plastic bags are one of the main sources of film microplastic contamination in urban and desert areas. Plastic woven bags without proper treatment and the widely-used plastic sand barriers for sand-fixing projects along the Taklamakan Desert highways might be the potential sources of flaky MPs. The fibers were mainly derived from the degradation of plastic carpets outside the city business districts, dust screens in peri-urban areas and plastic fiber sand barriers in the desert areas [24]. Most of the fragments and rubbers originated from household products and discarded broken car tires [25].

### 4.2. Characteristics of the MPs

#### 4.2.1. Abundance

The street dust in an industrialized and urbanized district of coastal Iran contained an average of about 900 MPs and 250 microrubbers (MRs) per 15 g sample [26]. The MPs were detected in street dust samples, ranging from 210 to 1658 items/10 g dust in Bushehr city, Iran [27]. The average MP abundance was 227.94 ± 91.37/100g of street dust samples in Chennai metropolitan city, India [28]. The microplastic concentrations ranged from ~0.5 mg/g (rural site) to 6 mg/g (city) in Australian urban road dust [29]. In contrast, the rates of MPs in the dust on the roads of Tokyo, Kumamoto and Okinawa are relatively small, at 230 ± 50, 96 ± 85 and 68 ± 77 items/kg, respectively [30]. The pollution level of MPs from street dust in southern Xinjiang, China, was in the middle when compared to similar international studies and may be related to the sample treatment, analysis methods or local urban pollution levels. The abundance of MPs in the street dust positively correlated with the urban population (Figure S3, R^2^ = 0.999, *p* = 0.003), which indicated that the MP abundance may be associated with the higher population density and traffic volume. The MPs on urban streets enter households or ventilated buildings as airborne particulates and may enter the body through respiration or ingestion, posing a risk to human health.

Agricultural plastic mulch has been widely used in agricultural production in arid regions. Mulching is an extremely important farming practice to maintain soil moisture levels in the Xinjiang Uygur Autonomous Region [31]. Previous studies have also shown that the longer the plastic film is used, the more serious the microplastic pollution will be [32]. In this study, the abundance of MPs in farmland was much lower than in Wuhan’s vegetable fields and vacant lots (mean concentration of 2020 items/kg) [33] and in the agricultural soils of southwestern China (mean 18,760 items/kg) [34] and northwestern China (ranged from 430 to 3410 items/kg) [35]. This was because of all samples were collected away from industrial areas, meaning the soils were not directly affected by personal care products and industrial MP inputs, implying that mulching film MPs were the most direct cause of agricultural soil microplastic contamination in southern Xinjiang, China. High levels of MPs in the soil have a significant impact on soil and soil–water relationships, such as the water-holding capacity, soil bulk density, soil microorganisms and soil structure.

The researchers found that the average MP abundance was 6.0 ± 15.4 items/kg in the Baden Jaran Desert, with 8.2 ± 17.9 items/kg on the edge of the desert where tourism activity occurs, which was much higher than the 0.9 ± 1.6 items/kg in non-tourist areas, indicating the potential contribution of tourism [36]. Due to the influence of human activities, the MP distribution along the desert road was uneven in the Taklamakan Desert and the abundance levels of MPs in the sand fixation and garbage disposal sites were much higher than in other sampling sites. Similarly, the MP abundance levels were affected by a variety of natural factors (e.g., temperature and sunlight) [37], as well as their own physicochemical properties (e.g., size and density) [38]. Due to the dry climate, long illumination time and strong ultraviolet rays in the desert region, many lightweight plastic products (such as plastic woven bags and sand barriers) rapidly age and constantly decompose under the wind–sand action, leading to the much higher abundance of MPs along the desert highway than in urban and rural areas [39]. After traveling long distances and due to atmospheric sedimentation, these MPs may finally enter urban areas, farmland and other areas of human activities and can pose a threat to human living environments and health.

#### 4.2.2. Shapes Distribution

The pollution from various anthropogenic activities has a significant impact on the shape distribution of MPs in different environments. The variety of MP shape categories was mainly related to the frequent and different human activities. In the environment, the plastic shapes are generally divided into pellets, fragments, foams, fibers and films. Among them, the fragments, fibers and films account for the main proportion in most studies.

Fragments accounted for 86% of the microplastic particles in Spanish agricultural soils [40]. Chai et al. [11] found that the fragments occupied the highest proportion of microplastics, accounting for 96.42%, in an e-waste dismantling area in Guangdong Province, China. Industrial and agricultural plastic packaging and domestic plastic waste materials break down and decompose under the action of external forces, which significantly contribute to the sources of fragmented MPs. Zhang et al. [34] investigated the MPs in the farmland soil of Dianchi Lake in Yunnan and found that the proportion of fibers was the highest (92%). Dris et al. [41] estimated that the annual atmospheric deposition of fibrous MPs in the populated areas of Paris equaled about 3–10 tons. The fibers were also the dominant shape found in soil samples from Washington, DC, and Nanjing and Wuxi, which were likely closely related to the increasing production of synthetic fibers. Li et al. [42] found that film particles dominated the cotton fields of Shihezi, Xinjiang. In Franconia and southeast Germany, the film particles occupy a considerable proportion of all MPs [43,44]. These films were possibly linked to plastic mulching and plastic packaging. These pellets were related to personal care products and the industrial masterbatching of products.

In this study, we found that the dominant fiber MPs were the most common shape in the urban street dust samples, which was consistent with previous reports [45]. A possible reason for the wide distribution of fiber MPs is the wide range of human production and living activities in urban environments. Film MPs were widely present, and were especially dominant in farmlands due to the degradation of residual plastic film materials [46]. However, the sources of thin-film MPs in urban and desert areas were mainly from discarded plastic bags, which were completely different from those in the farmland soils. These thin-film MPs may degrade again to form smaller MPs and spread to other areas with wind [47]. The flakes were only dominant in windproof and sand fixation engineering areas in desert areas, while the foams were only found in the urban streets, which indicated that the different living human activities led to a different shape category distribution of the MPs.

#### 4.2.3. Size Distribution

Measuring the sizes of MPs is somewhat complicated due to the definition and sensitivity of the extraction and analytical methods. In the sample pretreatment process, the particle sizes of the MPs separated from the samples were determined by the pore size of the sieve that was used to transfer and filter them. Researchers have found that MPs with small particle sizes have been widely observed in soil environments.

Small microfibers (<1 mm) were dominant in all samples of urban street dust. Dehghani et al. [21] found that MP sizes ranging from 250 to 500 µm were found in 10 street dust samples from the central district of Tehran. The proportion of small MPs (<500 µm) in facility agriculture was higher than those in bare farmlands and grasslands in the Qinghai–Tibet plateau [48]. Liu et al. [49] found that 48.79–59.81% MPs were <1 mm, mostly being fiber, fragment and film particles in the farmland soils in the suburbs of Shanghai, China. According to the literature reports, the abundance of MP pollution in facility farmland areas was the highest, followed by suburban agriculture, dominated by particle sizes of 0–0.50 mm. However, the pollution degree of agricultural MPs in the field was the lowest, dominated by particle sizes of 0~1 mm [50].

In our study, the MPs (<1 mm) accounted for the largest proportion, of which MPs measuring less than 500 µm dominated in all sample sites in southern Xinjiang, China, which was consistent with the size distribution of MPs reported previously. In the aging process of MPs, these MPs are broken down into smaller plastics after weathering, mechanical wear and sunlight, which may cause greater harm to the ecological environment.

### 4.3. Possible Environmental Risks and Risk Control

MPs are ubiquitous in the human environment. O’Connor et al. [51] found that smaller MPs were readily removed from the soil surface to deeper layers or groundwater caused by wind, surface runoff and infiltration. Carbery et al. [52] also suggested that smaller MPs can be ingested by organisms with lower trophic levels in the food chain and are more readily transferred into tissues than larger particles. Therefore, MPs with particle sizes < 500 µm can be very harmful to local organisms and ecosystems. Microplastic particles in street dust, which are less dense than mineral particles, have the potential to indirectly affect environments and ecosystems further afield through the contamination and transportation of street runoff and sewage sludge-based fertilizers [53]. Research has also shown that street dust MPs can enter the body through food and drinking water, as well as through respiration [54]. Luo et al. [55] found that sub-micron and even micron-grade plastic particles penetrate the roots of wheat and lettuce, flow through the system with water and nutrients and enter the edible parts of crops. The MPs in soil environments also have potential adverse impacts on human health through the food chain [56]. Little is known about the migration and transportation of MPs in arid regions, especially in desert regions. The MPs are tumbled and rubbed against the sand by strong, sustained winds and constantly produce smaller MPs, which may migrate to other areas with the wind and cause varying levels of MPs [57].

In view of the pollution characteristics of MPs in the terrestrial environment of southern Xinjiang and the potential ecological risks caused by MPs to the local biological security and diversity and soil micro-environment, a series of risk control measures should be taken. For example, the recovery and recycling efficiency of agricultural residual film should be accelerated and biodegradable film should be used instead of traditional polyethylene mulch film in agriculture. In cities with relatively concentrated populations, the use of plastic bags should be reduced, garbage sorting should be promoted and the recycling of discarded plastic waste should be strengthened. In desert areas with frequent human activities, specifically along the desert highways, the recycling and supervision of discarded plastic wastes should be strengthened and environmentally friendly windbreak and sand-fixing materials should be used to decrease the risk of microplastic pollution. At the same time, the local governments should formulate plastic product production, distribution, usage, recycling and disposal regulations and establish a long-term sustainable development mechanism in order to thoroughly resolve the problem of microplastic pollution.

This is the first study to systematically investigate the source, distribution, origin and trend of MPs in urban, farmland and desert areas in southern Xinjiang in China, and to present the basic pollution characteristics of MPs in this region, laying a foundation for further research on the ecological and environmental effects of MPs in arid areas. Although the existing studies have shown that MPs have a high pollution load in these areas and were characterized by their multiple types, high abundance and high risk, relevant investigations and research have only just started, and the overall pollution status of MPs in arid areas has not been displayed. Secondly, there is still a lack of widely accepted technical specifications for the sample collection, monitoring and analysis for MPs, resulting in the poor comparability of the survey results. Furthermore, it is difficult to carry out observations and research on micro-sized plastics (e.g., <100 microns). As a result, the pollution characteristics, distribution patterns and ecological impacts of smaller MPs in the terrestrial environment are still unclear. Therefore, it is necessary to organize and carry out comprehensive investigations of microplastic pollution at large spatial scales and to formulate systematic technical specifications for environmental monitoring, analyses and assessments and control standards for MPs in the terrestrial environment.

## 5. Conclusions

We reported on the extent of microplastic pollution in urban, farmland and desert areas along a highway in southern Xinjiang, China. Due to extensive human activities and meteorological conditions, microplastics are ubiquitous in southern Xinjiang and negatively affect the local ecological environment. In Korla, Alar and Hotan, the abundance of MPs is positively correlated with the urban population. In farmland, film materials with small particle sizes (<500 µm) were dominant in all sample sites. The distribution of MPs in southern Xinjiang was obviously affected by commerce, agriculture and transportation under the influence of extensive and diverse human activities. Based on the results from this survey, large-scale comprehensive research on the distribution and migration pattern of MPs in multimedia environments should be conducted and the ecological environmental hazards and human health risks in arid areas should be systematically evaluated based on the microplastic–environment behavior, microplastic ecotoxicity data and environmental exposure omics information in the future.

## Data Availability

This study does not report any data.

## Supplementary Materials

The following supporting information can be downloaded at: https://www.mdpi.com/article/10.3390/ijerph19158890/s1. Table S1: The latitude and longitude of the sampling point. Figure S1: Partial microplastic samples collected from the urban streets A ((a) Korla; (b) Alar; (c) Hotan), farmland soils (B) and deserts (C) along a highway in southern Xinjiang, China (Scale:10 mm). Figure S2: Examples of ATR-FTIR spectra of different microplastic samples from the cities, farmlands and deserts along the highways in southern Xinjiang, China: (a) flake; (b) film; (c,d) fragment; (e,f) fiber; (g) rubber. Figure S3: Correlation analysis of MP abundance levels and total populations in urban streets.
