# Supplementary material for: Microplastic Contamination in Urban, Farmland and Desert Environments along a Highway in Southern Xinjiang, China
Source: Int J Environ Res Public Health. 2022 Jul 22;19(15):8890. doi: 10.3390/ijerph19158890 (PMC9330657; doi:10.3390/ijerph19158890)
Supplement: Supplementary file 1 [file ijerph-19-08890-s001.zip › ijerph-1793928-supplementary.pdf]

Microplastic Contamination in Urban, Farmland and Desert Environments along a Highway in Southern Xinjiang, China

Wenfeng Li [1,2], Shuzhi Wang [1,2], Rehemanjiang Wufuer [1,2,*], Jia Duo [1,2] and Xiangliang Pan [2,3,*]

[1] National Engineering Technology Research Center for Desert-Oasis Ecological Construction, Xinjiang Institute of Ecology and Geography, Chinese Academy of Sciences, 818 South Beijing Road, Urumqi 830011, China; liwf86@ms.xjb.ac.cn (W.L.); wangshuzhi.1234@163.com (S.W.); duojia2017@ms.xjb.ac.cn (J.D.)
[2] Xinjiang Key Laboratory of Environmental Pollution and Bioremediation, Xinjiang Institute of Ecology and Geography, Chinese Academy of Sciences, Urumqi 830011, China
[3] Key Laboratory of Microbial Technology for Industrial Pollution Control of Zhejiang Province, College of Environment, Zhejiang University of Technology, Hangzhou 310014, China
* Correspondence: reheman319@ms.xjb.ac.cn (R.W.); panxiangliang@163.com (X.P.); Tel.: +86-991-7823-147 (R.W.)

Supplemental Materials

Table S1. The latitude and longitude of the sampling point

Sampling sites	East longitude	Northern latitude
S1	88.354112	42.471587
S2	87.043924	42.164337
S3	86.388255	42.085109
S4	86.175503	41.723228
S5	85.313010	41.525938
S6	83.078241	41.415412
S7	82.376130	41.096750
S8	81.285256	40.545725
S9	81.202698	40.357010
S10	81.094723	40.314287
S11	80.986576	40.021272
S12	80.986555	39.533306
S13	80.949862	39.129111
S14	80.985117	38.761680
S15	80.943832	38.405716
S16	80.617118	38.097889
S17	80.522275	37.807784
S18	80.318685	37.537159
S18	79.971714	37.274394
S20	79.913993	37.111547
S21	79.433052	37.110478
S22	78.312968	37.350792

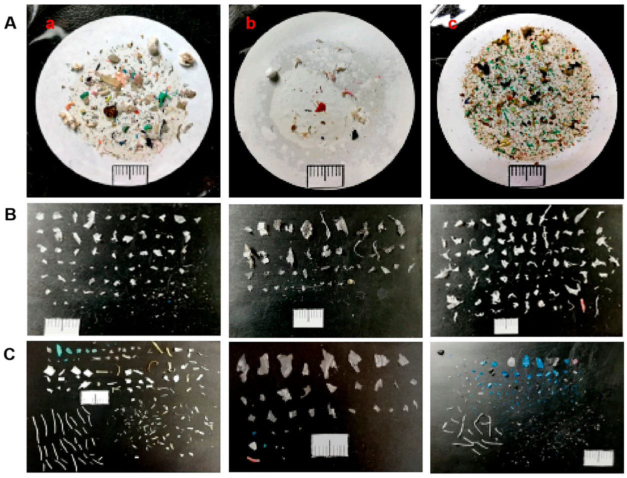

Figure S1 Partial microplastic samples collected from the urban streets A (a: Korla; b: Alar; c: Hotan), farmland soils (B) and deserts (C) along a highway in southern Xinjiang, China (Scale:10 mm).

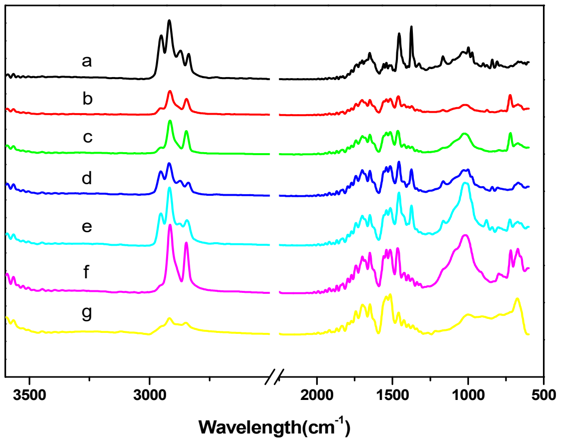

Figure S2 Examples of ATR-FTIR spectras of different microplastic samples from the cities, farmlands and deserts along the highways in the southern Xinjiang, China; a, flake; b, film; c, d, fragment; e, f, fiber; g, rubber.

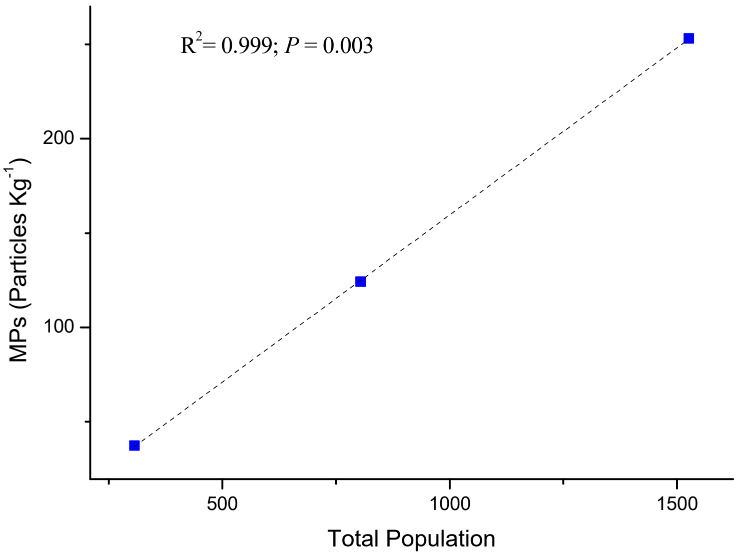

Figure S3 Correlation analysis of MPs abundance and total population in urban streets.

According to the statistics yearbook of the Xinjiang Region (2019) and the Xinjiang Construction Corps (2019), the Bayingoll Mongol Autonomous Prefecture where Korla is located has a population of ~1.24 million, Aral has a population of ~372,000 and the population of Hotan district is ~2.53 million.
